# Recent advances and perspectives in efforts to reduce the production and application cost of microbial flocculants

**DOI:** 10.1186/s40643-021-00405-2

**Published:** 2021-06-14

**Authors:** Cong Liu, Di Sun, Jiawen Liu, Jingrong Zhu, Weijie Liu

**Affiliations:** Jiangsu Key Laboratory of Phylogenomics & Comparative Genomics, School of Life Science, Jiangsu Normal University, No.101, Shanghai road, Tongshan New District, Xuzhou, 221116 Jiangsu China

**Keywords:** Microbial flocculants, Bioflocculant-producing strain, Polysaccharide flocculant, Alternative medium, Application, Extraction

## Abstract

Microbial flocculants are macromolecular substances produced by microorganisms. Due to its non-toxic, harmless, and biodegradable advantages, microbial flocculants have been widely used in various industrial fields, such as wastewater treatment, microalgae harvest, activated sludge dewatering, heavy metal ion adsorption, and nanoparticle synthesis, especially in the post-treatment process of fermentation with high safety requirement. However, compared with the traditional inorganic flocculants and organic polymeric flocculants, the high production cost is the main bottleneck that restricts the large-scale production and application of microbial flocculants. To reduce the production cost of microbial flocculant, a series of efforts have been carried out and some exciting research progresses have been achieved. This paper summarized the research advances in the last decade, including the screening of high-yield strains and the construction of genetically engineered strains, search of cheap alternative medium, the extraction and preservation methods, microbial flocculants production as an incidental product of other biological processes, combined use of traditional flocculant and microbial flocculant, and the production of microbial flocculant promoted by inducer. Moreover, this paper prospects the future research directions to further reduce the production cost of microbial flocculants, thereby promoting the industrial production and large-scale application of microbial flocculants.

## Introduction

Flocculant is a kind of agent that can reduce or eliminate the colloid stability of dispersed particles in water, and make the dispersed particles coagulate and flocculate into aggregates (Kaarmukhilnilavan et al. 2020; Salehizadeh and Shojaosadati 2001; Salehizadeh et al. 2018). Therefore, flocculants are widely used in various industrial fields, such as wastewater treatment, mining, food processing, and post-treatment of fermentation (Liu et al. 2015d, 2019; Salehizadeh and Yan 2014; Salehizadeh et al. 2018; Shahadat et al. 2017), among which the demand for flocculants is the largest in the field of wastewater treatment. At present, flocculants are mainly divided into inorganic flocculants, organic polymeric flocculants, and bioflocculants (Salehizadeh and Shojaosadati 2001; Salehizadeh and Yan 2014). Among them, inorganic flocculants are mainly represented by aluminum sulfate, poly-aluminum chloride, ferric chloride, and ferrous sulfate (Okaiyeto et al. 2016; Salehizadeh and Yan 2014). However, ferric salt is corrosive, and the accumulation of aluminum salt in human body is directly related to Alzheimer’s disease (Campbell 2002). Organic polymeric flocculants are represented by polyacrylamide derivatives and polyethyleneimine (Salehizadeh and Shojaosadati 2001). However, polyacrylamide derivatives are difficult to be degraded, and its degraded monomer acrylamide exhibits a strong biological toxicity (Salehizadeh and Yan 2014). Therefore, the application of inorganic flocculants and organic polymeric flocculants in industrial fields with high safety requirement is greatly limited (Okaiyeto et al. 2016). Bioflocculant is a class of biological macromolecular substances with flocculating activity obtained from biological sources (Salehizadeh et al. 2018), including plant sources, such as polysaccharides extracted from cactus; animal sources, such as bioflocculant obtained from *Ruditapes philippinarum* (Mu et al. 2018, 2019); and microbial sources, such as microbial flocculants extracted from fermentation broth of microorganism (Shahadat et al. 2017). Among them, the studies on microbial flocculants are the most concerned.

Microbial flocculants are mainly composed of polysaccharides, proteins produced by microorganisms in the process of fermentation, and nucleic acids released mainly by cell lysis (Liu et al. 2010; Tang et al. 2014a). Compared with inorganic flocculants and organic polymeric flocculants, microbial flocculants have the characteristics of biodegradation, non-toxic and harmless, no secondary pollution (Chaisorn et al. 2016; Liu et al. 2015d). Therefore, microbial flocculants show good safety advantages in food processing and fermentation post-treatment processes (Ndikubwimana et al. 2014). For example, during the production of biodiesel using microalgae cells, the concentration cost of microalgae cells accounts for 30–50% of the production cost of biodiesel (Liu et al. 2015d; Wan et al. 2013). Flocculation is an effective method to reduce the cost of microalgae harvest (Liu et al. 2015d, 2017a; Roy and Mohanty 2020). However, the safety of flocculants is a key consideration, because in addition to producing biodiesel, microalgae cells can also be used for the extraction of microalgae polysaccharides or the production of animal feeds (Bernaerts et al. 2018; Markou and Nerantzis 2013). Using inorganic flocculants or organic polymeric flocculants to flocculate microalgae has adverse effects on the quality of biodiesel and the production of high value-added products from microalgae cells (Liu et al. 2017a; Wan et al. 2013). Therefore, the research on the application of bioflocculant in microalgae concentration has attracted more and more interests (Bukhari et al. 2020; Lei et al. 2015; Li et al. 2016b; Liu et al. 2015a, 2017a; Ndikubwimana et al. 2016; Sarang and Nerurkar 2020; Sivasankar et al. 2020; Sun et al. 2015a, b; Wan et al. 2013; Wang et al. 2015; Xu et al. 2018b; Zou et al. 2018). With the increasing attention to environmental protection and safety, microbial flocculants have been used in many industrial fields, such as wastewater treatment (Agunbiade et al. 2017; Chaisorn et al. 2016; Guo et al. 2013; Li et al. 2013; Liu et al. 2016b, 2019, 2020; Luo et al. 2014, 2016; Ma et al. 2020; Pu et al. 2014, 2018; Sajayan et al. 2017; Zhang et al. 2021; Zhong et al. 2020), nanoparticle synthesis (Dlamini et al. 2019, 2020; Manivasagan et al. 2015; Muthulakshmi et al. 2017, 2019; Rasulov et al. 2016a, b; Sathiyanarayanan et al. 2013; Zaki et al. 2014), heavy metal ion adsorption (Agunbiade et al. 2019; Ayangbenro et al. 2019; Cao et al. 2015; Chen et al. 2016; Feng et al. 2013; Gomaa 2012; Guo 2015; Guo and Chen 2017a; Guo and Yu 2014; Huang et al. 2019; Li et al. 2016a; Pathak et al. 2017; Pu et al. 2020; Subudhi et al. 2016; Vimala et al. 2020; Yan et al. 2020), activated sludge dewatering (Guo and Ma 2015; Guo et al. 2015b, d; Liu et al. 2014; Yang et al. 2012, 2017), dye decolorization (Wang et al. 2020; Xia et al. 2018), pathogen removal from water (Zhao et al. 2013), and membrane fouling reduction (Deng et al. 2015). Therefore, microbial flocculants gradually replace the traditional inorganic and organic polymeric flocculants which will become an inevitable trend. In Table 1, we summarize the industrial applications of microbial flocculants. At present, compared with the traditional flocculants, the market share of microbial flocculant products is still relatively low due to its high production cost resulted by fermentation medium and fermentation process (Liu et al. 2016b, 2017a,^ 2019^,^ 2020^; Salehizadeh and Yan 2014), which restricts the large-scale application of microbial flocculants.

**Table 1:** Application fields of microbial flocculants

Applications	Strains or sources	Dosage	Descriptions	Ref.
Biomass harvest	*Bacillus agaradhaerens*	8 mg/L	Microalgae harvest, FR of 80.63% to *Chlorella minutissima*	Liu et al. (2015b)
	*Enterobacter aerogenes*	13.5 mg/L	Microalgae harvest, FR of 91.68 to 97.21% to *Microcystis aeruginosa*	Xu et al. (2018b)
	*Pseudomonas aeruginosa*	1.75 mg/L	Microalgae harvest, FR of 100% to *Microcystis aeruginosa*	Sun et al. (2015a, b)
	*Cobetia marina*	20 mg/L	Microalgae harvest, FR of 92.7% to *Chlorella vulgaris*	Lei et al. (2015)
	*Shinella albus*	30 mg/L	Microalgae harvest, FR of 85.65% to *Chlorella vulgaris*	Li et al. (2016b)
	*Streptomyces* sp.	5 g/L	Microalgae harvest, FR of 99.18% to *Nannochloropsis*	Sivasankar et al. (2020)
	*Bacillus marisflavi*	100 mg/L	Microalgae harvest, FR of 90% to *Chlorella vulgaris*	Bukhari et al. (2020)
	*Cellulosimicrobium cellulans*	250 mL/L	Microalgae harvest, FR of 99.0% to *Chlamydomonas reinhardtii*	Liu et al. (2015b)
	*Bacillus licheniformis*	2.5 mL/L	Microalgae harvest, FR of 99% to *Desmodesmus brasiliensis*	Ndikubwimana et al. (2016)
	*Bacillus amyloliquefaciens*	243 mg/L	Microalgae harvest, FR of 87.98% to *Microcystis aeruginosa*	Sun et al. (2015a, b)
	*Citrobacter* sp.	12.7 mg/L	Microalgae harvest, FR of 95% to *Microcystis aeruginosa*	Xu et al. (2017)
	*Pseudomonas boreopolis*	80 mg/L	Microalgae harvest, FR of 95.7% to *Scenedesmus abundans*	Guo et al. (2018a, b)
	*Solibacillus silvestris*	1.1 g/L	Microalgae harvest, FR of 85.7% to *Nannochloropsis oceanica*	Wan et al. (2013)
	*Cloacibacterium normanense*	5.8 mg/g	Yeast harvest, FR of 74.07% to *Yarrowia lipolytica*	Yellapu et al. (2019)
	*Paecilomyces* sp.	700 mg/L	Yeast harvest, FR of 95% to *Trichosporon fermentans*	Qiao et al. (2019)
Metal ion removal	*Turicibacter sanguinis*	500 mg/L	Remove 86.1% arsenite from solution	Cao et al. (2015)
	*Stenotrophomonas maltophilia*	40 mg/L	Remove 81.4% Cd^2+^ from solution	Chen et al. (2016)
	*Bacillus megaterium*	0.005%	Remove 99.2% arsenite from solution	Guo and Chen (2017a)
	*Pseudomonas koreensis*	1 g/L	Remove 51.2% Cd^2+^, 52.5% Cr^6+^ and 80.5% Pb^2+^ from solution	Ayangbenro et al. (2019)
	*Bacillus megaterium*	1.25 g/L	Remove 82.64% Pb^2+^, 51.82% Zn^2+^ and 33% Ni^2+^ from solution	Pu et al. (2020)
	*Achromobacter xylosoxidans*	1 g/L	Absorb over 95% Pb^2+^ from solution	Subudhi et al. (2016)
	*Enterococcus faecalis*, *Proteus mirabilis*, *Lysini* sp.	28 mg/L	Adsorb 95% Cu^2+^, 72% Zn^2+^, 58% Hg^2+^, 92% Cd^2+^ from solution	Vimala et al. (2020)
	*Rhodococcus erythropolis*	0.035%	Remove 96.9% Cu^2+^ from solution	Guo (2015)
	*Terrabacter* sp.	500 mg/L	Remove 77.7% Fe^3+^, 74.8% Al^3+^, 61.9% Mn^2+^, 57.6% Zn^2+^ from dairy wastewater	Agunbiade et al. (2019)
	From activated sludge	6 mg/L	Remove 98.5% of Pb^2+^ from solution	Yan et al. (2020)
	*Pseudomonas aeruginosa*	100 ppm	Absorb 79.7% Pb^2+^, 79.9% Cd^2+^, 72.9% As^5+^ and 80.6% Zn^2+^ from solution	Gomaa (2012)
	*Paenibacillus elgii*	1 g/L	Remove 53% Cu^2+^, 49% Co^2+^, 60% Pb^2+^, 72% Al^3+^ from solution	Li et al. (2013)
	*Pseudomonas aeruginosastrain*	20 mg/L	Remove 79.29% Ni^2+^ from solution	Pathak et al. (2017)
	*Bacillus sphaericus* and *Rhizobium radiobacter*	28 mg/L	Remove 92.95% Al^3+^ of river water	Li et al. (2016a)
	*Paenibacillus polymyxa*	0.006%	Remove 99.85% Pb^2+^ from solution	Feng et al. (2013)
Sludge dewatering	*Rhodococcus erythropolis*	10.5 g/kg	DS and SRF of sludge appeared as 24.1% and 3.0 × 10^12^ m/kg	Guo and Chen (2017b)
	From pre-treated sludge	1.6 g/L	DS and SRF of the sludge reached 22.5% and 3.4 × 10^12^ m/kg	Guo and Ma (2015)
	*Paenibacillus polymyxa*	1.5 g/L	DS and SRF of activated sludge reached 20.8% and 3.9 × 10^12^ m/kg	Guo et al. (2015d)
	*Klebsiella* sp.	6 g/kg	DS and SRF of sludge reached 17.5% and 3.36 × 10^12^ m/kg	Yang et al. (2012)
	*Azotobacter chroococcum*	80 mg/L	Dewatering of coal waste slurry, FR of 83% to coal waste slurry	Yang et al. (2017)
Wastewater treatment	*Bacillus agaradhaerens*	6 mg/L	Remove 93.1% turbidity from straw ash-washing wastewater	Liu et al. (2020)
	*Diaphorobacter nitroreducens*	831 mg/L	Remove 96% turbidity, 79% COD, 59% lignin, 63% sugar from pulping wastewater	Zhong et al. (2020)
	*Bacillus cereus*	10 mg/L	Reduce 62% COD, 55% BOD, 76% TDS, 74% TSS from distillery effluent	Sajayan et al. (2017)
	*Bacillus subtilis*	60 mg/L	Remove 27.3% SS of palm oil mill effluent	Chaisorn et al. (2016)
	From pre-treated sludge	20 mg/L	Remove 45.2% COD, 41.8% ammonium, 74.6% turbidity from swine wastewater	Guo and Ma (2015)
	*Pseudomonas veronii*	2.83 mg/L	Remove 92.51% turbidity from ash flushing wastewater	Liu et al. (2016a, b)
	*Bacillus agaradhaerens*	9 mg/L	Remove 92.35% turbidity from mineral processing wastewater	Liu et al. (2019)
	*Paenibacillus polymyxa*	30 mg/L	Remove 49.5% COD and 74.6% turbidity from potato starch wastewater	Guo et al. (2015a)
	*Terrabacter* sp.	500 mg/L	Remove 54.1% COD, 63.3% BOD, 66.6% SS, 75.6% nitrate, 89.7% turbidity of dairy wastewater	Agunbiade et al. (2019)
	*Enterobacter* sp.	1000 mg/L	Remove 85% chroma and 52% SS of fracturing flowback water	Ma et al. (2020)
	*Bacillus fusiformis*	110 mg/L	Remove 22.7% total nitrogen, 28.5% COD, 20.4% colority from tannery wastewater	Zhao et al. (2016)
	*Arthrobacter humicola*	800 mg/L	Remove 65.7% COD, 63.5% BOD, 55.7% SS, 71.4% nitrate, 81.3% turbidity of sewage wastewater	Agunbiade et al. (2017)
	*Alteromonas* sp.	200 mg/L	Remove 98.5% congo red, 97.9% direct black, 72.3% methylene blue from dye wastewater	Chen et al. (2017a)
	*Aspergillus niger*	3.78 mg/L	Remove 91.15% COD and 60.22% turbidity from potato starch wastewater	Pu et al. (2018)
	*Klebsiella variicola*	333 mg/L	Achieve 84.7% decolorization efficiency to methylene blue solution	Xia et al. (2018)
	*Rhodococcus* sp.	24 mg/L	Remove 87.9% COD, 86.9% ammonium and 94.8% turbidity from swine wastewater	Guo et al. (2013)
	*Paenibacillus elgii*	30 mL/L	Remove 68% COD, 83% turbidity, 88% color from real wastewater	Li et al. (2013)
	*Rhizopus* sp.	0.1 mL/L	Remove 54.09% COD and 92.11% turbidity from potato starch wastewater	Pu et al. (2014)
	*Aspergillus niger*	35 mg/L	Remove 63% turbidity of river water	Aljuboori et al. (2014)
	*Klebsiella* sp.	5 mg/L	Remove 53.27% sulfamethoxazole in domestic wastewater	Xing et al. (2013)
	*Klebsiella pneumoniae*	44 mg/L	Remove 72% TSS from raw wastewater	Nie et al. (2011)
	*Sphingomonas yabuuchiae*	50 mg/L	Remove 87% estrone, 92% estradiol, 88% ethinylestradiol, 96% estriol from estrogen solution	Zhong et al. (2014)
	*Oceanobacillus polygoni*	4 g/L	Remove 46.49% SS and 91.08% turbidity from tannery wastewater	Li et al. (2017)
	*Bacillus salmalaya*	60 mg/L	Remove 81.3% Zn^2+^, 78.6% As, 77.9% Pb^2+^, 76.1% Cu^2+^, 68.7% Cd^2+^ from synthetic wastewater	Tawila et al. (2019)
	*Bacillus* sp.	2%	Remove 82.8% color, 92.5% COD, 73.6% TSS, 81.9% Cl^−^ from dyeing wastewater	Bisht and Lal (2019)
	*Haloplanus vescus*	150 mg/L	Removed 81.86 COD and 95.07% chroma from dye wastewater	Zhong et al. (2016)
	*Cellulomonas taurus*		Removed 71.05% COD, 18.22 ammonia nitrogen from pig farm wastewater	Zhang et al. (2021)
	*Bacillus* sp.	20 mg/L	Remove 47% COD and 89% TSS from municipal wastewater	Kanmani and Yuvapriya (2018)
Nanoparticle synthesis	*Bacillus* sp.		Bioflocculant diffused cellulose in AgNO_3_ solution, generated nanoparticles AgNPs	Muthulakshmi et al. (2017)
	*Streptomyces* sp.		Add bioflocculant to AgNO_3_ solution, produced silver nanoparticles	Manivasagan et al. (2015)
	*Bradyrhizobium japonicum*		Add bioflocculant to AgNO_3_ solution, produced nanoparticles AgCl-NPs	Rasulov et al. (2016a)
	*Bacillus* sp.		Bioflocculant diffused cellulose in CuSO_4_ solution, obtained nanoparticles (CuNPs)	Muthulakshmi et al. (2019)
	*Alcalegenis faecalis*	2.5 g/L	Add bioflocculant in CuSO_4_ solution, synthesized nanoparticles CuNPs	Dlamini et al. (2020)
	*Azotobacter chroococcum*		Bioflocculant exposed to AgNO_3_ solution, produced nanoparticles AgCl-NPs	Rasulov et al. (2016b)
	*Bacillus subtilis*	5%	Add AgNO_3_ to bioflocculant solution, generated nanoparticles AgNPs	Sathiyanarayanan et al. (2013)
	*Bacillus mojavensis*	10%	Add AgNO_3_ to bioflocculant solution, synthesize nanoparticles AgNPs	Zaki et al. (2014)
Other applications	*Bacillus subtilis*	0.1–1 g/L	Exhibited antibacterial, antioxidant, and anti-inflammatory potential	Giri et al. (2019)
	*Stenotrophomonas maltophilia*	0.001–1 g/L	Used as hemostasis agent	Zhao et al. (2017)
	*Paenibacillus jamilae*	100 mg/L	Used as hemostasis in clinical settings	Zhong et al. (2018)
	*Enterococcus faecalis*	11.57 mg/L	Recover graphene oxide, FR over 90% to graphene oxide in water	Xu et al. (2018a)

*FR* flocculating rate, *SS* suspended solids, *DS* dry solids, *SRF* specific resistance to filtration, *COD* chemical oxygen demand, *BOD* biological oxygen demand, *TSS* total suspended solids

To reduce the production and application cost of microbial flocculants, a series of efforts and strategies have been carried out. Especially in the last 10 years, some exciting research progresses have been achieved. This paper summarizes the latest research advances on the screening of high-yield strains, the exploitation of cheap alternative medium, the construction of genetic engineering strain, the extraction and preservation methods, and other strategies to reduce the production and application cost of microbial flocculants, and put forward the new development trend and research direction of microbial flocculant, thereby promoting its large-scale production and applications.

## Efforts to reduce the production and application cost of microbial flocculants

### Screening of high-yield strains

One of the effective strategies to reduce the production cost of microbial flocculants is to screen high-yield strains and optimize their fermentation conditions, so it has always been a hot research work in this field. At present, the reported microbial flocculants producing strains mainly include bacteria (Salehizadeh and Yan 2014), actinomycetes (Nwodo et al. 2014), fungi (Aljuboori et al. 2015), and algae (Tiwari et al. 2015), among which bacterial strains are the most common source for the production of microbial flocculants. So far, more than 100 strains have been reported to be able to produce microbial flocculants, some of which show high yield or good application prospects, including *Lipomyces starkeyi* U9 (Yu et al. 2020b), *Alteromonas* sp. (Chen et al. 2017a), *Bacillus agaradhaerens* C9 (Liu et al. 2015a, 2017a,^ 2019^), *Solibacillus silvestris* W01 (Wan et al. 2013), *Paenibacillus elgii* B69 (Li et al. 2013), *Agrobacterium* sp. M-503 (Li et al. 2010), *Klebsiella oxytoca* GS-4-08 (Yu et al. 2016), *Paenibacillus mucilaginosus* (Tang et al. 2014a), *Achromobacter* sp. TERI-IASST N (Subudhi et al. 2014), *Bacillus marisflavi* NA8 (Bukhari et al. 2020), and *Bacillus subtilis* MSBN17 (Sathiyanarayanan et al. 2013). Tables 2 and 3 summarize the fermentation conditions and yields of the reported strains. Furthermore, screening strains that can produce microbial flocculant under low nutritional condition is also an effective strategy to reduce the production cost of microbial flocculants. For example, *Chryseobacterium daeguense* W6 can produce microbial flocculant at low nutrient condition. In the early stage of fermentation, the strain W6 grew rapidly and the flocculating activity of fermentation broth was low; however, in the late stage of fermentation, the cell lysis of strain W6 occurred rapidly and released microbial flocculant product (Liu et al. 2010). In addition, the flocculating activity of some microbial flocculants depends on the activation of metal ions. Therefore, selecting the strains that can produce microbial flocculant independent of metal ions is conducive to reducing the application cost of microbial flocculants and avoiding the secondary pollution caused by the addition of metal ions (Liu et al. 2010; Tang et al. 2014b; Yin et al. 2014). The reported strains producing microbial flocculant independent of metal ions are listed in Table 3. The dependence of flocculating activity on metal ions generally depends on the composition of microbial flocculants. Most positively charged microbial flocculants do not require metal ions and their flocculating activity is mainly achieved by charge neutralization with negatively charged suspended particles (Liu et al. 2015c; Mohammed and Dagang 2019b).

**Table 2:** Microbial flocculant producing strains and their composition, extract method, and yield

Strains	Carbon and nitrogen sources (g/L)	Components	Extracts	Yields (g/L)	Ref.
*Bacillus agaradhaerens*	Glucose 10, YE 10	Ps 65.4%, Pr 4.7%, NA 1.6%	EP	4.65	Liu et al. (2015a)
*Streptomyces* sp.	Palm jaggery 18.73, YE 2.07	Ps	EP		Manivasagan et al. (2015)
*Bradyrhizobium japonicum*	Sucrose 25, YE 2.5	Ps	EP		Rasulov et al. (2016a)
*Streptomyces* sp.	YE 2.5, palm jiggery 20, NH_4_NO_2_ 1	Ps 86.9%, Pr 12.8%	AP, IEC, GFC	4.94	Sivasankar et al. (2020)
*Alcaligenes faecalis*	Maltose 20, rea 1.2	Ps 88.6%, Pr 9.5%	EP	4.00	Maliehe et al. (2019)
*Bacillus* sp.	Glucose 20, (NH_4_)_2_SO_4_ 0.3, urea 0.5, YE 0.5	Ps 76%, Pr 14%	EP	1.60	Okaiyeto et al. (2015b)
*Alteromonas* sp.	Glucose 30, wheat flour 1.5	Ps 69.6%, Pr 21.5%	EP	11.18	Chen et al. (2017a)
*Bacillus licheniformis*	Sucrose 10, YE 1, urea 1	Ps 89%, Pr 11%	EP	2.93	Xiong et al. (2010)
*Bacillus thuringiensis*	Beef extract 3.0, peptone 10.0	Ps	EP	20.19	Wang et al. (2011)
*Lipomyces starkeyi*	Glucose 100, polypeptone 7.0	Ps	EP	53.50	Yu et al. (2020b)
*Streptomyces*, *Cellulomonas*	Sucrose 16, peptone 1.5	Ps 34.4%, Pr 18.5%	EP	4.45	Nwodo et al. (2014)
*Chryseobacterium daeguense*	Glucose 1, Tryptone 2	Ps 13%, Pr 32%, NA 6.8%	EP	0.89	Liu et al. (2010)
*Bacillus mojavensis*	l-Glutamic acid 20, NH_4_Cl 7	Ps 98.4%, Pr 1.6%	EP	5.20	Elkady et al. (2011)
*Aspergillus flavus*	Sucrose 30, peptone 3	Ps 69.7%, Pr 28.5%	EP	0.40	Aljuboori et al. (2013)
*Solibacillus silvestris*	Maltose 1.9, YE 11	Ps 75.1%, Pr 24.9%	AP	0.40	Wan et al. (2013)
*Paenibacillus elgii*	Sucrose 51.3, peptone 6.7, YE 0.47	Ps	EP	25.63	Li et al. (2013)
*Virgibacillus*	Glucose 20, urea 0.5, YE 0.5, (NH_4_)_2_SO_4_ 0.2	Ps	EP	2.43	Sekelwa et al. (2013)
*Agrobacterium* sp.	Sucrose 20, YE 0.5, urea 0.5, (NH_4_)_2_SO_4_ 0.2	Ps 97%, Pr 3.0%	EP, IEC, GFC	14.90	Li et al. (2010)
*Achromobacter* sp.	Sucrose 10, urea 10	Ps 57%, Pr 13%	EP	10.50	Subudhi et al. (2014)
*Enterobacter* sp.	Glucose 10, NaNO_3_ 1	Ps 91.7%, Pr 1.8%	EP, CTAB-p	0.066	Tang et al. (2014b)
*Methylobacterium* sp. and *Actinobacterium* sp.	Glucose 20, (NH_4_)_2_SO_4_ 0.2, urea 0.2, YE 0.5		EP	8.20	Luvuyo et al. (2013)
*Pseudomonas aeruginosa*	Sucrose 5, glucose 2, maltose 2, YE 5, NH_4_Cl 1.5	Ps 71.7%, Pr 16.4%, NA 2.1%	EP	3.58	Sun et al. (2015a)
*Paenibacillus mucilaginosus*	Sucrose 20, YE 0.5	Ps	EP, IEC, GFC	1.33	Tang et al. (2014a)

*YE* yeast extract, *Ps* polysaccharide, *Pr* protein, *NA* nucleic acid, *EP* ethanol precipitate, *AP* acetone precipitate, *IEC* Ion exchange chromatography, *GFC* Gel filtration chromatography, *CTAB-p* cetyltrimethyl ammonium bromide precipitate

**Table 3:** Correlation between composition of microbial flocculant with its thermal stability, metal ion dependence and flocculating mechanism

Strains	Compositions	Stability (°C)	Metal ions	Mechanisms	Ref.
*Bacillus agaradhaerens*	Ps 65.4%, Pr 4.7%, NA 1.6%	3–63	Ca^2+^-independent		Liu et al. (2015a)
*Bacillus aryabhattai*	Glycoprotein	40–80	Activated by Ca^2+^		Abd El-Salam et al. (2017)
*Bacillus subtilis*	Ps 88.3%, Pr 10.1%	10–100	Activated by Ca^2+^		Giri et al. (2015)
*Bacillus megaterium*	Ps 85.5%, Pr 14.3%	10–120	Ca^2+^-independent	Bridging, charge neutralization	Guo and Chen (2017a)
*Rhodococcus erythropolis*	Ps 95.6%, Pr 4.4%	10–120	Ca^2+^-independent	Bridging, charge neutralization	Guo et al. (2015c)
*Diaphorobacter nitroreducens*	Ps 73.9%, Pr 24.1%	20–80	Activated by Ca^2+^, Mg^2+^	Charge neutralization	Zhong et al. (2020)
*Klebsiella* sp.	Ps 84.6%, Pr 11.1%	30–100		Bridging mechanism	Liu et al. (2013)
*Bacillus cereus*	Ps	30–100			Sajayan et al. (2017)
*Aspergillus flavus*	Ps 69.7%, Pr 28.5%	5–45	Cation-independent	Charge neutralization	Aljuboori et al. (2015)
*Sphingomonas yabuuchiae*	Ps 91%, Pr 9%	20–80	Cation-independent		Tang et al. (2014b)
*Paenibacillus jamilae*	Ps 89.2%, Pr 6.3%	10–100			Zhong et al. (2018)
*Bacillus pumilus*	Ps 83.1%, Pr 6%	50–100	Required Ba^2+^	Bridging mechanism	Maliehe et al. (2016)
*Chryseobacterium daeguense*	Ps 13.1%, Pr 32.4%, NA 6.8%	instability	Cation-independent	Attachment and bridging neutralization	Liu et al. (2015c)
*Klebsiella* sp.	Ps 84.6%, Pr 6.1%	up to 115	Cation-independent	Bridging mechanism	Yin et al. (2014)
*Bacillus marisflavi*	Ps 74%, Pr 25%, NA 1%	10–100	Cation-dependent		Bukhari et al. (2020)
*Paenibacillus polymyxa*	Ps 96.2%	30–110	Enhanced by Ca^2+^	Adsorption, bridging, charge neutralization	Guo et al. (2015a)
*Bacillus toyonensis*	Ps 77.8%, Pr 11.5%	50–80	Increased by Mn^2+^		Okaiyeto et al. (2015a)
*Bacillus amyloliquefaciens*	Ps 57.12%		Improved by Ca^2+^	Charge neutralization	Sun et al. (2015b)
*Bacillus pumilus*	Ps 75.4%, Pr 5.3%, NA 15.4%	up to 100	Enhanced by Ca^2+^, Mg^2+^, Mn^2+^		Makapela et al. (2016)
*Klebsiella variicola*	Ps 81.8%, Pr 15.9%	20–100	Increased by Ca^2+^, Fe^2+^, Mg^2+^, Mn^2+^	Bridging, charge neutralization	Xia et al. (2018)
*Chlamydomonas reinhardtii*	Ps 48%, Pr 42%, lipids 8.7%		Enhanced by Ca^2+^		Zhu et al. (2012)
*Bacillus megaterium*	Ps 78.5%, Pr 9.2%, others 12.3%	20–100	Activated by Ca^2+^, inhibited by Al^3+^, Fe^3+^	Bridging mechanisms	Pu et al. (2020)
*Pseudomonas aeruginosa*	Ps 89%, Pr 27%	100	Improved by Ca^2+^, K^+^, Na^+^, Zn^2+^, Mg^2+^, Cu^2+^; inhibited by Fe^3+^, Al^3+^		Gomaa (2012)

*Ps* polysaccharide, *Pr* protein, *NA* nucleic acid

### Construction of genetically engineered strains

The construction of genetically engineered strains is an efficient approach to improve the yield of microbial flocculant and further reduce its production cost. However, only a few microbial flocculant producing strains have been genetically engineered. In *Bacillus licheniformis* CGMCC2876, a polysaccharide-related gene cluster *epsA-O* and regulatory genes *sinR* and *slrR* were identified through genome sequencing and comparative genomics analysis (Chen et al. 2017b). Both EpsE and EpsF are glucosyltransferases involved in the conversion of UDP-glucose into polysaccharide. EpsD is a glucuronyltransferase that utilizes UDP-glucuronic acid as substrate. Overexpression of *epsDEF* in *B. licheniformis* CGMCC2876 enhanced the flocculating activity by 90% and increased the yield of polysaccharide flocculant by 27.8% compared to the original strain (Chen et al. 2017b). Overexpression of the UDP-glucose pyrophosphorylase gene in *B. licheniformis* CGMCC2876 not only increased the flocculating activity of the recombinant strain by 71%, but also increased yield by 13.3% compared to the original strain (Chen et al. 2017b). EpsB plays a critical role in the biosynthesis of polysaccharide in *B. licheniformis*. Overexpressing *epsB* increased the flocculating activity to 9612.75 U/mL and the yield to 10.26 g/L, which enhanced by 224% and 36.62%, respectively, compared to the original strain (Liu et al. 2017b). Moreover, the tandem expression of phosphoglucomutase (*pgcA*) and UTP-glucose-1-phosphate uridylyltransferase (*gtaB1*) was able to increase the yield by 20.77% and overexpression of *epsA* was able to enhance the yield by 23.70% compared to the original strain (Liu et al. 2017b). In addition, in *Lipomyces starkeyi* V9, overexpression of UDP-glucose dehydrogenase gene was able to improve the exopolysaccharide yield of from 53.5 to 62.1 g/L (Yu et al. 2020b).

The lack of mature genetic operation system and complex synthetic regulation mechanism restricts the construction of genetic engineering bacteria of most microbial flocculant producing strains. For the strains with immature genetic operation system or unclear regulation mechanism, it is a good choice to use random mutation technology to improve the production of microbial flocculants. A high-yield mutant of *Bacillus cereus* was obtained based on mutation effect of MeV protons and successfully increased the flocculating activity of microbial flocculant by more than 20% (Yang et al. 2007). Random mutation technology generally needs to establish efficient screening models of high-yielding mutants, which helps to reduce workload and improve breeding efficiency. For most strains producing polysaccharide flocculant, according to the principle that macromolecular polysaccharide can adsorb Congo red dye, the high-yield mutants can be preliminarily judged based on the strains with redder colony color on the screening medium plate added with Congo red dye, thereby improving the screening efficiency of target mutants.

### Search for cheap alternative medium

The production of microbial flocculant with cheap substitute substrate is not only beneficial to decrease the production cost of microbial flocculants, but also to realize the resource utilization of solid wastes or wastewaters. High concentration organic wastewater is rich in organic substance, which can be used as fermentation carbon source or nitrogen source to cheaply produce microbial flocculants, such as potato starch wastewater (Guo et al. 2015a, d, 2018b; Pu et al. 2014, 2018), brewery wastewater (Ma et al. 2020), corn ethanol wastewater (Xia et al. 2018), swine wastewater (Guo and Chen 2017a), palm oil mill effluent (Aljuboori et al. 2014; Bukhari et al. 2020; Hassimi et al. 2020), livestock wastewater (Peng et al. 2014), ramie biodegumming wastewater (Zhong et al. 2020), phenol-containing wastewater (Chen et al. 2016), and chromotropic acid wastewater (Zhong et al. 2014). In Table 4, we summarize the inexpensive wastes or wastewaters that have been selected as low-cost alternative fermentation medium to produce microbial flocculants.

**Table 4:** Production of microbial flocculants using cheap wastes or waste waters as alternative medium

Strains	Fermentation mediums	Yields (g/L)	Ref.
*Stenotrophomonas maltophilia*	Phenol-containing wastewater with 800 mg/L phenol, dissolved oxygen concentration 2%	4.99	Chen et al. (2016)
*Bacillus megaterium*	(mg/L) Swine wastewater contained COD 1065, ammonia 828, and total phosphorus 26	3.11	Guo and Chen, (2017a)
*Rhodococcus erythropolis*	(g/L) Rice stover hydrolyzate, K_2_HPO_4_ 4, KH_2_PO_4_ 2, MgSO_4_ 0.2, NaCl 0.1, urea 0.5, yeast extract 0.5	2.37	Guo et al. (2015c)
*Bacillus agaradhaerens*	(g/L) Chicken feather 40, glucose 16, K_2_HPO_4_ 1.4, KH_2_PO_4_ 0.7, NaCl 0.5, MgSO_4_7H_2_O 0.1, Na_2_CO_3_ 10	2.50	Liu et al. (2020)
*Diaphorobacter nitroreducens*	Ramie biodegumming wastewater of 1500 mg/L COD used as fermentation medium	3.86	Zhong et al. (2020)
*Pseudomonas* sp.	Rice straw biomass of 0.5% in mineral salt medium	1.75	Qi et al. (2019)
*Bacillus velezensis*	Palm oil mill effluent medium	2.03	Hassimi et al. (2020)
*Klebsiella oxytoca*	Acetonitrile 1 g/L, glucose 8 g/L, Na_2_HPO_4_ 50 mM, KH_2_PO_4_ 100 mM, MgSO_4_ 1 mM, CaCl_2_ 0.1 mM	4.60	Fan et al. (2019)
*Sphingomonas yabuuchiae*	(g/L) Chromotropic acid wastewater, K_2_HPO_4_ 5, KH_2_PO_4_ 2, MgSO_4_ 0.2, urea 0.5, yeast extract 0.5	9.71	Zhong et al. (2014)
*Pseudomonas veronii*	(g/L) Peanut hull hydrolyzate yeast extract 3, K_2_HPO_4_ 0.6, MgSO_4_.7H_2_O 0.1	3.39	Liu et al. (2016b)
*Bacillus agaradhaerens*	(g/L) Kitchen waste 40, Na_2_CO_3_ 10	6.92	Liu et al. (2019)
*Bacillus agaradhaerens*	(g/L) Rice bran 20, yeast extract 3, Na_2_CO_3_ 20	12.94	Liu et al. (2017a)
*Aspergillus flavus*	Hydrolysate of chicken viscera	6.00	Mohammed and Dagang (2019a)
*Bacillus marisflavi* NA8	Enzymatic hydrolysate of palm oil mill effluent	9.72	Bukhari et al. (2020)
*Klebsiella pneumoniae*	Starch processing industrial wastewater	1.12	Joshi et al. (2017)
*Paenibacillus polymyxa*	(g/L) Potato starch wastewater, K_2_HPO_4_ 4, KH_2_PO_4_ 2, MgSO_4_ 0.2, NaCl 0.1, urea 2.0	0.81	Guo et al. (2015a)
*Enterobacter* sp.	(g/L) Brewery wastewater (COD 1.48), glucose 8.94	1.27	Ma et al. (2020)
*Cellulosimicrobium cellulans*	(g/L) Dry corn stover of 20, yeast extract of 3, Na_2_CO_3_ 0.4	4.75	Liu et al. (2015d)
*Rhodococcus erythropolis*	(g/L) Potato starch wastewater, K_2_HPO_4_ 4, KH_2_PO_4_ 2, MgSO_4_ 0.2, NaCl 0.1, urea 2	0.97	Guo et al. (2018b)
*Citrobacter* sp.	(g/L) Wet biomass of *Microcystis aeruginosa* 10 and glucose 10	3.40	Xu et al. (2017)
*Aspergillus niger*	(g/L) Potato starch wastewater (COD 5.9), glucose 20, urea 0.2	0.82	Pu et al. (2018)
*Klebsiella variicola*	(g/L) Corn ethanol wastewater, K_2_HPO_4_ 5, KH_2_PO_4_ 2, MgSO_4_ 0.2, NaCl 0.1	3.08	Xia et al. (2018)
*Rhodococcus* sp.	Alkaline-thermal treated sludge 100 g/L	4.20	Guo et al. (2013)
*Bacillus subtilis*	(g/L) Palm jaggery 20, yeast extract 2.5, NH_4_NO_2_ 1.0, MgCl_2_ 0.2, K_2_HPO_4_ 5, NaCl 0.1	13.42	Sathiyanarayanan et al. (2013)
*Rhizopus* sp.	(g/L) Potato starch wastewater with COD of 1.6, urea 0.3, KH_2_PO_4_ 0.04	0.69	Pu et al. (2014)
*Aspergillus niger*	(g/L) Palm oil mill effluent of TOC 10; glutamic acid 7.92; MgSO_4_ 0.5; KCl 0.5; FeSO_4_ 0.01; K_2_HPO_4_ 1.0	2.73	Aljuboori et al. (2014)
*Rhodococcus erythropolis*	Excess sludge from municipal wastewater treatment and livestock wastewater	1.60	Peng et al. (2014)
*Schizophyllum commune*	(g/L) Hydrolysates of rice hulls supplemented with yeast extract 3, KH_2_PO_4_ 0.5, MgSO_4_.7H_2_O 0.25	1.30	Shu and Hsu (2011)
*Ochrobactium ciceri*	(g/L) Corn stover hydrolysates, K_2_HPO_4_ 5, KH_2_PO_4_ 2, MgSO_4_ 0.2, NaCl 0.1, urea 0.5, yeast extract 0.5	3.80	Ma et al. (2020)
*Enterococcus faecalis*, *Proteus mirabilis*, *Lysini bacillus* sp.	Hydrolyzed wheat bran extract, hydrolyzed peanut hull extract and 0.1% MgSO_4_	5.01	Vimala et al. (2020)

*COD* chemical oxygen demand, *TOC* total organic carbon

**Fig. 1:**
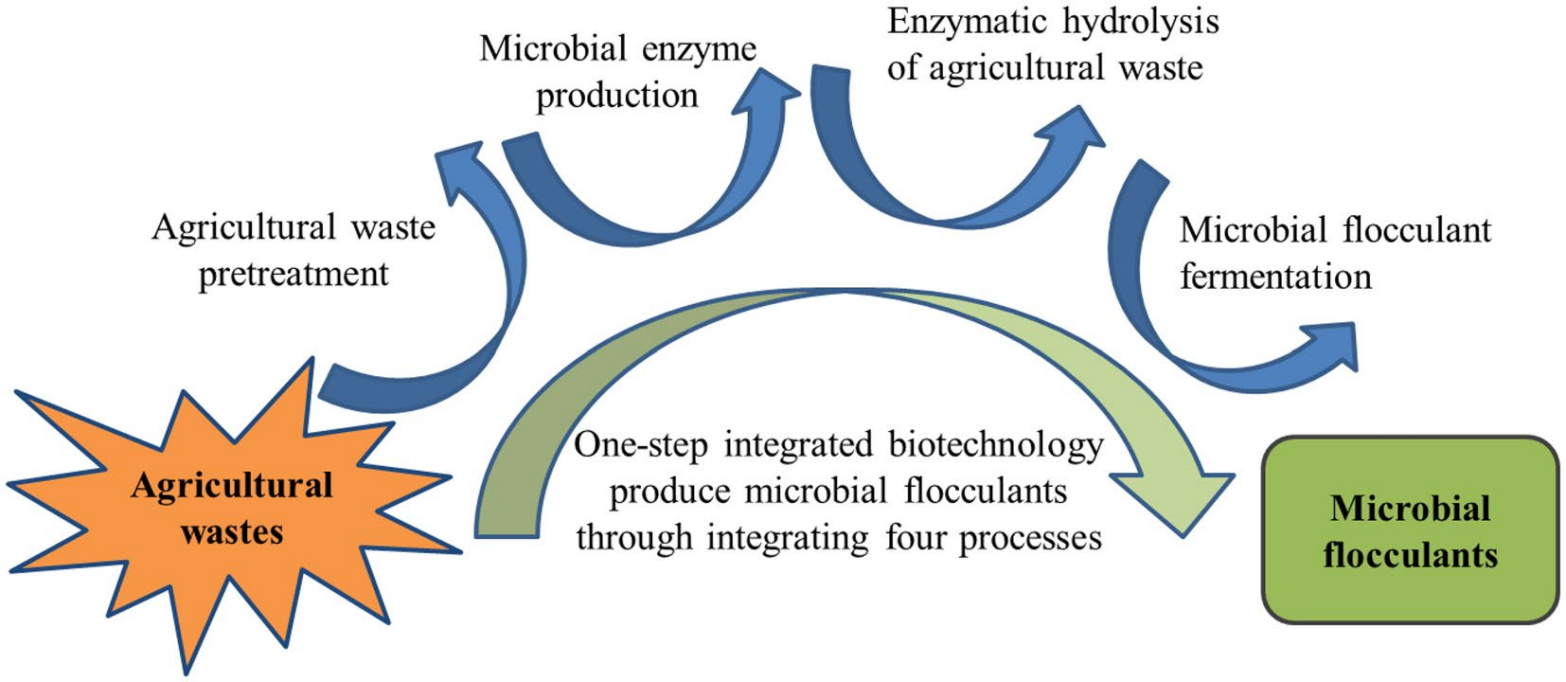
Microbial flocculant produced by one-step integrated biotechnology

Lignocellulosic agricultural wastes, such as corn straw, corncob, peanut hull, and rice bran, can be decomposed into reducing sugars, and then converted into other high value-added products through microbial fermentation (Monlau et al. 2014). Therefore, how to efficiently convert these agricultural wastes into valuable products and reduce environmental pollution is one of the current research hotspots (Liu et al. 2015d). To cut down the production cost of microbial flocculants, the hydrolysates of agricultural waste obtained from hot sulfuric acid hydrolysis were used as the carbon source of fermentation medium. For examples, using the hydrolysate of corn straw as the fermentation carbon source of *Rhodococcus erythropolis* to produce microbial flocculant, the yield reached 2.4 g/L (Guo et al. 2015c); the microbial flocculant yield of *Ochrobacium ciceri* W2 reached 6.2 g/L using the hot acidic hydrolysate of rice husk as the carbon source (Wang et al. 2014) and the yield of 3.39 g/L microbial flocculant was achieved when peanut hull hydrolyzate was used as carbon source of *Pseudomonas veronii* L918 (Liu et al. 2016b). However, the hot acidic hydrolyzate of agricultural wastes requires the pH neutralization using calcium hydroxide before the subsequent fermentation processes (Guo et al. 2015c; Wang et al. 2013), which increases the operation difficulty and the production cost (Liu et al. 2015d). And the hydrolyzates of agricultural wastes always contain toxic by-products, such as phenolic compounds and furan derivatives (Monlau et al. 2014), which inhibit the microbial activities in the fermentation processes (Mussatto and Roberto 2004), and remain in the microbial flocculant products. Therefore, strains that can secrete lignocellulolytic enzymes and simultaneously produce microbial flocculants through directly degrading lignocellulosic biomasses are of academic and practical interests. For example, *Cellulosimicrobium cellulans* L804 can secrete cellulase and xylanase, and directly convert untreated corn straw into microbial flocculant by one-step integrated biotechnology which integrates the processes of agricultural waste pretreatment, microbial enzyme production, the enzymatic hydrolysis of agricultural waste, and microbial flocculant fermentation (Fig. 1), with a yield of 4.75 g/L, and exhibits a good flocculating activity to microalgae *Chlamydomonas reinhardtii* and *Chlorella minutissima* (Liu et al. 2015d). Compared with the traditional fermentation using pure sugar as carbon source, one-step integrated biotechnology using agricultural waste as carbon source can efficiently decrease the production cost of microbial flocculants; compared with the hydrolysate of agricultural waste as carbon source, it can avoid the toxic by-products produced in the process of hot acid hydrolysis (Monlau et al. 2014; Liu et al. 2015d). However, the optimal fermentation condition (pH 9.0) of *C. cellulans* L804 for microbial flocculant production was different from the optimal condition (pH 6.0) of self-secreted cellulase and xylanase. The activities at fermentation condition (pH 9.0) of these two enzymes were only half of their optimal conditions at pH 6.0, which limited the efficiency of enzymatic hydrolysis of corn straw in one-step integrated biotechnology by *C. cellulans* L804 (Liu et al. 2015d). To solve the condition divergence of enzyme activity and fermentation of microbial flocculant, an alkaline-tolerant *Bacillus agaradhaerens* C9 was isolated from alkaline lake water (Liu et al. 2015a). The lignocellulose degrading enzyme of *B. agaradhaerens* C9 showed high enzyme activity in the range of pH 9.0–10.8, which was same as the optimal fermentation condition for producing polysaccharide flocculant (Liu et al. 2017a). Therefore, this strain can directly and efficiently convert untreated agricultural wastes (such as corn straw, rice bran, and peanut shell) into microbial flocculant in one-step integrated biotechnology. Moreover, alkaline fermentation condition was able to promote the expansion of lignocellulose structure, and increase the specific surface area of enzymatic hydrolysis, thereby improving the conversion efficiency from agricultural waste into microbial flocculant, and the highest yield of 12.94 g/L was achieved, which showed a flocculating rate of 91.05% to *Chlorella minutissima* (Liu et al. 2017a). *Pseudomonas boreopolis* G22 was found to be able to secrete xylanase and simultaneously produce microbial flocculant. Thus, *P. boreopolis* G22 was used as a fermentation strain in one-step integrated biotechnology to convert grass lignocelluloses (agave, corn stover, Miscanthus, and wheat bran) into microbial flocculant. The yield reached 3.75 mg/g dry biomass, and the flocculation rate of obtained microbial flocculant to *Scenedesmus abundans* reached 95.7% (Guo et al. 2018a, b).

At present, most of the studies are focused on the alternative carbon source, but few on the alternative nitrogen source. Feather is the solid waste of poultry processing plants (Kshetri et al. 2019). In addition to some fine feathers are used as filling materials of clothes, mattresses, and comforters, huge amount of feather wastes are discarded, causing environmental pollution, because feather waste is difficult to be degraded due to the strong hydrophobicity of keratin (Gao et al. 2014). Feather waste can be decomposed by keratinase producing strain and used as nitrogen source to produce microbial flocculants. *B. agaradhaerens* C9 is a bifunctional strain that can simultaneously produce keratinase and microbial flocculant. Therefore, *B. agaradhaerens* C9 can utilize feather waste as nitrogen source to produce microbial flocculant, which exhibited a good flocculating activity to straw ash-washing wastewater with low-density and high pH property, and the yield of 2.5 g/L was obtained (Liu et al. 2020). In addition, kitchen waste is a semi-solid waste produced mainly by families, canteens and restaurants (Karthikeyan et al. 2018). It contains various organic substances such as starch, fat, protein, cellulose, pectin, and inorganic salt, which provide complete nutritional requirements for microorganisms to produce microbial flocculants. The strains that can directly convert kitchen waste into microbial flocculants generally need to produce a variety of degrading enzymes to decompose macromolecular substances in kitchen waste. *B. agaradhaerens* C9 was found to be able to produce multiple degradation enzymes including amylase, protease, lipase, cellulase, xylanase, and pectinase, achieving resourceful utilization of kitchen waste to produce microbial flocculants, which was successfully applied in the treatment of mining wastewater, and the yield reached 6.92 g/L (Liu et al. 2019).

### Direct extraction from natural substances

Biofilm is an aggregate of bacterial cells, which are encapsulated by self-secreted polysaccharides, proteins, and nucleic acids (Flemming et al. 2016). Some bacterial biofilms contain macromolecular substances with flocculation activity, so microbial flocculants can be extracted from some bacterial biofilms. For example, microbial flocculant was extracted from periphytic biofilm, and the yield reached 491.8 mg/g biofilm (Sun et al. 2018). Activated sludge contains a large amount of organic matters secreted by microorganisms in the process of wastewater treatment. It can be directly used as the fermentation medium for microbial flocculant production after heating pretreatment, alkali, or acid pretreatment (Guo et al. 2013, 2014). In addition, activated sludge is an aggregate composed of bacterial cells and extracellular polymeric substances, including polysaccharides and proteins produced by microbial metabolism, which has the potential to directly extract microbial flocculants. At present, microbial flocculants have been successfully extracted from activated sludge and applied in the wastewater treatment (Liu et al. 2009; Sun et al. 2012; Yan et al. 2020; Zhang et al. 2013), which not only reduces the production cost of microbial flocculants, but also realizes the resourceful utilization of activated sludge. However, the quality of extracted microbial flocculant is greatly affected by the source of activated sludge. Low purity limits its application in industrial fields with high safety requirement. Therefore, the microbial flocculants extracted from activated sludge are mainly used in fields with low purity requirement, such as wastewater treatment (Liu et al. 2009).

### Exploration of the extraction and preservation methods

The cost of extraction and purification accounts for 30%–50% of the total production cost of microbial flocculants. Therefore, exploring efficient extraction methods or changing the application way is able to reduce the production cost of microbial flocculants. According to the purity requirement of microbial flocculants, the main extraction methods include organic reagent precipitation, gel adsorption, and high-performance liquid chromatography purification (Li et al. 2010; Sivasankar et al. 2020; Tang et al. 2014a), among which organic reagent precipitation is the most frequently used extraction method (Aljuboori et al. 2013; Chen et al. 2017b; Elkady et al. 2011; Li et al. 2013; Liu et al. 2015a, d, 2016b, 2020; Luvuyo et al. 2013; Manivasagan et al. 2015; Sekelwa et al. 2013; Subudhi et al. 2014; Sun et al. 2015b; Tang et al. 2014a, b; Wan et al. 2013; Wang et al. 2011; Xiong et al. 2010). Table 2 summarizes the extraction methods of some microbial flocculants. During the extraction process, microbial flocculant products generally lose 30–50% flocculating activity (Liu et al. 2015a), and the extracted solid microbial flocculant needs to be dissolved in the solution before use to increase the dispersion of microbial flocculant in the solution and the contact probability with the suspended solid particles. Most of the microbial flocculants are macromolecular substances (Liu et al. 2015d), which are difficult to be dissolved, thereby increasing the operation difficulty. Therefore, under the premise of comprehensive consideration of transportation cost, it is also an ideal choice to directly use the liquid fermentation broth of microbial flocculants. Furthermore, improving the stability and prolonging the shelf life of microbial flocculant are also an effective strategy to reduce the application cost of microbial flocculants. However, a few studies on the stability of microbial flocculants were carried out. Previous study found that beta‑glucosidase secreted by *Bacillus licheniformis* could degrade self-produced polysaccharide flocculant. Therefore, the deletion of beta‑glucosidase synthesis gene improved the stability of microbial flocculant in fermentation broth of *B. licheniformis* (Chen et al. 2017c). The stability analysis of microbial flocculant in liquid fermentation broth produced from *B. agaradhaerens* C9 showed that its flocculating activity could maintain above 85% at 4 °C for more than 3 months (Liu et al. 2015a). In addition, correlation between composition of microbial flocculant and its thermal stability is listed in Table 4, suggesting that the thermal stability of polysaccharide flocculant is generally higher than that of protein flocculant, and the thermal stability of microbial flocculant depends on the relative contents of polysaccharide and protein (Chaisorn et al. 2016; Chen et al. 2017c). Therefore, to improve the stability of microbial flocculants and reduce its application cost, the strains that can produce microbial flocculant with high stability should be selected as industrial strains.

### Microbial flocculant production as an incidental product of other biological processes

It is an effective strategy to reduce the production cost through producing microbial flocculant as an incidental product of other biological processes. Other microbial metabolites or other biological processes provide cost compensation for the production of microbial flocculants (Table 5). For examples, in the fermentation process of biological hydrogen production by *Bacillus* sp. XF-56 (Liu et al. 2015b), *Pantoea agglomerans* BH18 (Liu et al. 2016a) and *Enterobacter aerogenes* (Xu et al. 2018b), microbial flocculants are produced as an incidental product. The yield of microbial flocculant reached 3.6 g/L during the hydrogen production by *E. aerogenes* (Xu et al. 2018b). *Klebsiella oxytoca* GS-4-08 produced 4.92–5.21 g/L microbial flocculant during the process of nitriles degradation (Yu et al. 2020a). And during the fermentation process for methane production by *Methanosarcina spelaei* RK-23, the flocculating activity of fermentation broth reached 95.6% (Zhao et al. 2020).

**Table 5:** Microbial flocculant production as an incidental product of other biological processes

Strains	Bioprocesses	Yields or flocculating activity	Ref
*Enterobacter aerogenes*	Biohydrogen production	Bioflocculant yield 3.6 g/L and biohydrogen 35 mL H_2_/g dry weight algal biomass	Xu et al. (2018b)
*Klebsiella oxytoca*	Nitriles degradation	Bioflocculant yield was 4.92–5.21 g/L	Yu et al. (2020a)
*Bacillus* sp.	Production of hydrogen	Hydrogen yield was 1.79 mol H_2_/mol glucose and flocculating activity was 98.6%	Liu et al. (2015b)
*Pantoea agglomerans*	Production of hydrogen	Hydrogen yield was 1.55 mol H_2_/mol glucose and flocculating activity was 83.7%	Liu et al. (2016a)
*Methanosarcina spelaei*	Methane production	Methane yield was 17.4 mmol methane/mol acetate and flocculating activity was 95.6%	Zhao et al. (2020)

### Combined use of traditional flocculant and microbial flocculant

Combined use of microbial flocculants and traditional inorganic flocculants or organic polymeric flocculants is an useful way to reduce the application cost of microbial flocculants, because this method can play a synergistic role between them, which is conducive to reducing the consumption of microbial flocculants (Guo and Chen 2017b; Guo et al. 2015b; Huang et al. 2013; Li et al. 2014; Zhao et al. 2012). For examples, compared with microbial flocculants and aluminum sulfate alone, dual-coagulant of microbial flocculants and aluminum sulfate obviously improved the flocculating efficiency to Kaolin-humic acid solution (Bo et al. 2011). The combination of polysaccharide flocculant and poly-aluminum chloride significantly enhanced the removal efficiency of dissolved organic carbon in low-temperature drinking water and accelerate the growth rate of flocs (Huang et al. 2015b). The composite flocculant composed of microbial flocculant and aluminum salt showed a good application effect on synthetic dye wastewater, which can improve the floc size under acidic conditions and increase the formation speed of flocs under neutral or alkaline conditions (Huang et al. 2015a). After grafting with acrylamide chains, microbial flocculant produced by *Bacillus pumilus* JX860616 exhibited a good flocculating activity to domestic wastewater, the removal of chemical oxygen demand (COD), biochemical oxygen demand (BOD), total nitrogen, and total phosphorous reached 98%, 54%, 53%, and 57%, respectively (Ngema et al. 2020). When microbial flocculant produced by *Paenibacillus polymyxa* or conventional polyacrylamide was used independently to dewater the activated sludge, specific resistance to filtration (SRF) decreased by 65.5% and 71.7%, and dry solids (DS) increased to 20.8 and 24.2%, respectively. Interestingly, the sludge dewatering by the complex of microbial flocculant and polyacrylamide was improved with SRF decreased by 81.4% and DS increased to 28.4% (Guo et al. 2015d). The composite of microbial flocculant and poly(acrylamide [2-(methacryloyloxy)ethyl]-trimethylammonium chloride) (P(AM-DMC)) exhibited a good dewater ability to activated sludge, and DS and SRF appeared as 29.9% and 2.2 × 10^12^ m/kg, which is significantly higher than DS 21.7% and SRF 3.6 × 10^12^ m/kg of sludge treated by microbial flocculant alone (Guo et al. 2015b). The harvesting efficiency of *Chlorella regularis* achieved a level of 96.77% with the combination use of microbial flocculant, AlCl_3_, and coagulant aid (CaCl_2_), which is obviously better than the flocculation activity of microbial flocculant (52%), Chemical Flocculant (49%), and coagulant aid (66%) alone (Zhang et al. 2016). In addition, the compound flocculant composed of microbial flocculant, aluminum sulfate, and poly-aluminum chloride can increase the treatment of synthetic dyeing wastewater (Huang et al. 2014).

### Microbial flocculant production promoted by inducer

Some quorum-sensing signal molecules or chemical inducers can promote the fermentation production of microbial flocculants. It was found that the addition of quorum-sensing signal molecule *n*-hexanoyl-homoserine lactone (C6-HSL) into the fermentation medium of *Agrobacterium tumefaciens* strain F2 can promote the production of microbial flocculant. The yield of polysaccharide flocculant was enhanced by 1.75 times, and the flocculation activity was increased by 10% when the concentration of C6-HSL was 0.45 μM (Huang et al. 2014). Furthermore, *Agrobacterium tumefaciens* F2 was found to be able to secrete *N*-3-oxo-octanoyl-homoserine lactone (3-oxo-C8HSL), a microbial quorum-sensing signaling molecule of the *N*-acyl-homoserine lactone (AHL) class. The addition of 0.22 µM exogenous 3-oxo-C8HSL increased the production of exopolysaccharide flocculant by 1.55 times and the flocculation efficiency increased by 10.96% (Wu et al. 2015). In addition, a rotifer secretion produced from the species *Philodina erythrophthalma* was found to be able to significantly enhance the flocculability of *Brevundimonas vesicularis* LW13 and *Bacillus cereus* LW19, and promote the formation of microbial aggregation and floc (Ding et al. 2017).

## Perspectives in future research

### Develop microbial flocculants with wide application scope

At present, most of the reported microbial flocculants are only analyzed for the flocculating effect to 1–3 kinds of suspension sample, and the flocculation mechanism of different microbial flocculants is generally different when they flocculate the suspended solid particles (Table 3), mainly including charge neutralization, sweeping flocculation, and bridging flocculation. However, the existing research results indicate that most of the reported microbial flocculants are not able to flocculate all kinds of wastewater or cell suspension. Only a few microbial flocculants can simultaneously flocculate printing and dyeing wastewater, mining wastewater, and algae cell suspension. In general, the flocculation effect of microbial flocculants depends on different flocculation mechanisms and the surface charge, structural features, and particle size of suspended particles. The application scope of polysaccharide flocculant depending on metal ions is relatively wider, because the flocculation mechanism of polysaccharide flocculant is mostly metal ion-mediated bridging effect (Table 3), sometimes accompanied by charge neutralization effect, thus forming macromolecular bridging network to capture suspended particles (Xia et al. 2018). Therefore, in the future research, more attention should be paid to the screening of microbial flocculant producing bacteria with a wide application scope.

### Construction of genetic engineering strains at genetic level

Future research should focus on improving the production of microbial flocculant by constructing genetically engineered strains. Until now, only a few strains have been genetically modified, including *Bacillus licheniformis* CGMCC2876 (Chen et al. 2017b, c; Liu et al. 2017b) and *Lipomyces starkeyi* U9 (Yu et al. 2020b). This is mainly due to the complex structure and large molecular weight of microbial flocculants, which lead to the complex gene regulation of microbial flocculant synthesis. For example, in *Bacillus subtilis*, polysaccharides are synthesized and regulated by gene clusters composed of dozens of genes (Branda et al. 2005). *Bacillus* genus contains different species, most of which can synthesize macromolecular polysaccharides, but not all the polysaccharides synthesized by *Bacillus* have flocculating activity, indicating that the synthesis of microbial flocculant is very complex, which limits the construction of genetically engineered strain. Future research can focus on identifying functional genes through comparative genomics; for example, by comparing the gene clusters responsible for the synthesis of polysaccharides with and without flocculating activity, thereby identifying the functional genes regulating the synthesis of polysaccharide flocculants. In addition, it is also an ideal strategy for overexpression of key functional genes related to the synthesis of microbial flocculants, or deletion of functional genes that inhibit the synthesis of microbial flocculants and the genes related to microbial flocculant degrading enzyme. For example, in *Bacillus subtilis* 168, the core transcription factor SinR is a key inhibitor of polysaccharide synthesis gene cluster (Chu et al. 2006). By deleting *sinR* gene, the synthesis of polysaccharide can be significantly up-regulated.

### Obtain high-yield strains using genome shuffling

**Fig. 2:**
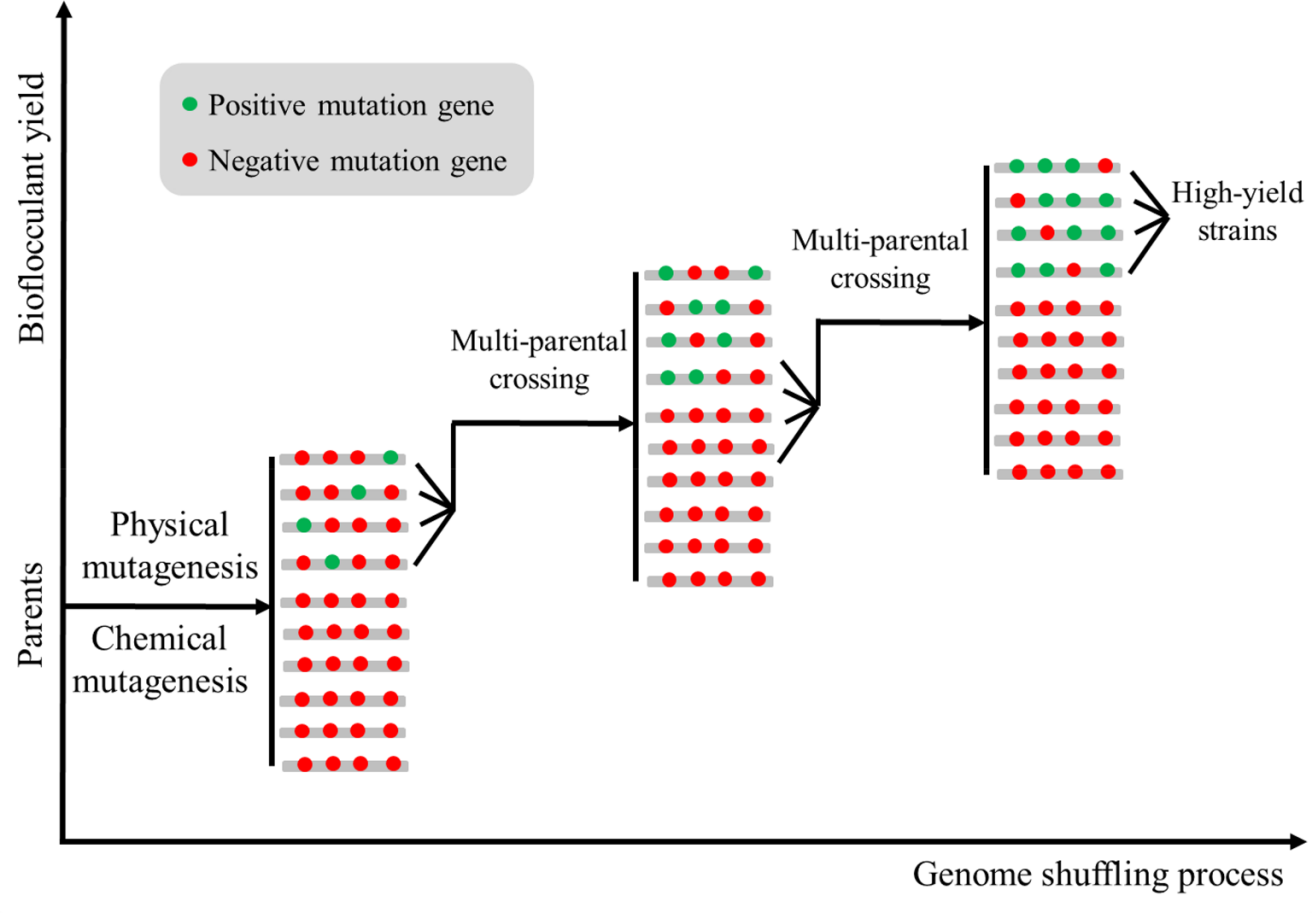
Flowchart of microbial flocculant producing bacteria modified by genome shuffling

To solve the problem of complex and unclear synthesis mechanism of microbial flocculants, genome shuffling is also a good choice for most microbial flocculant producing strains without mature genetic operation system (Zhang et al. 2002). Genome shuffling can complete the recombination at different sites of the whole genome and integrate a variety of excellent phenotypes of the parent plants, which makes up for the defects of the classical physical and chemical mutation breeding to a large extent; the mutant used for genome reorganization comes from the same parent, which is easier to cross to form a stable phenotype; compared with genetic engineering breeding technology, genome shuffling technology does not need to know the whole-genome sequence data and metabolic regulatory network information. Therefore, in recent years, genome shuffling technology has been widely used to improve the yield of microbial metabolites or enhance the adaptability of microorganisms to adverse environment (Gong et al. 2009). Therefore, the microbial flocculant producing strain can be modified using genome shuffling in the future researches according to the technical process shown in Fig. 2.

### Combination use of bifunctional strains to convert organic wastes to microbial flocculants

The bifunctional bacteria that simultaneously secrete degrading enzymes and produce microbial flocculants can directly convert macromolecular organic wastes into microbial flocculants without pretreatment process, such as using corn straw, corncob, or kitchen waste as the substrate of fermentation medium. However, the composition and structure of these macromolecular organic wastes are complex, and a variety of degradation enzymes are required to improve their degradation and conversion efficiency. It is difficult for single strain to provide a complete enzyme system. Therefore, it is also a good research direction to use multiple microbial flocculant producing bacteria that produce different degradation enzymes to synergistically utilize complex organic wastes, such as kitchen wastes and agricultural wastes.

### Exploration of cheap culture medium

At present, the exploration of cheap carbon source has gained research progresses to some extent, but it is still lack of the search for cheap alternative nitrogen source and phosphorus source. Future studies should continue to explore cheap alternative fermentation medium to decrease the production cost of microbial flocculants.

### Scale-up fermentation and application process

At present, most studies on microbial flocculant are at the laboratory level, and the large-scale fermentation production and application are still relatively lacking. Future research should focus on the parameter optimization during the process of technological scale-up test.

### Combined use of microbial flocculant with traditional flocculant or adsorbent

Using traditional flocculants or adsorbents as flocculant aid can reduce the dosage of microbial flocculants and improve the flocculation efficiency. For example, the combined use of microbial flocculant and coal fly ash or activated carbon is able to play the adsorption role of fly ash and activated carbon to dye molecules in dyeing wastewater or metal ions in heavy metal wastewater. At the same time, with the help of microbial flocculants to accelerate flocculation sedimentation, the treatment efficiency of wastewater can be enhanced.

### Explore cheap extraction methods and improve the stability of microbial flocculants

At present, the extraction of microbial flocculants is mainly achieved by organic reagent precipitation method, which accounts for more than 30% of the total production cost. Moreover, after extraction, the activity of microbial flocculant loses obviously. Therefore, future research needs to explore cheaper extraction methods. In addition, if liquid fermentation broth is directly used as liquid microbial flocculant, the extraction cost can be avoided. Future research should focus on improving the storage stability of liquid microbial flocculants, for example, by knocking out the microbial flocculants degrading enzyme gene in engineering strains, so as to improve the storage stability of liquid microbial flocculants.

## Conclusions

Microbial flocculants will gradually replace inorganic flocculants and organic polymeric flocculants in more and more industrial fields due to the advantages of environmental friendly and efficient characteristics. Microbial flocculants have been successfully applied in the industrial fields with high safety requirement. However, the high production cost is still the main bottleneck problem that limits the large-scale production and application of microbial flocculants. The application scale and scope of microbial flocculants in the future depend on further reducing their production and application cost. In the future studies, the screening and construction of efficient functional strains, cheap culture medium, new fermentation production and application strategy, cheap extraction, and storage strategy are the key research directions.

## Data Availability

Not applicable.

## Abbreviations

AHL: *N*-Acyl-homoserine lactone
AP: Acetone precipitate
BOD: Biological oxygen demand
COD: Chemical oxygen demand
CTAB-p: Cetyltrimethyl ammonium bromide precipitate
C6-HSL: *N*-Hexanoyl-homoserine lactone
3-oxo-C8HSL: *N*-3-Oxo-octanoyl-homoserine lactone
DS: Dry solids
EP: Ethanol precipitate
FR: Flocculating rate
GFC: Gel filtration chromatography
IEC: Ion exchange chromatography
NA: Nucleic acid
Ps: Polysaccharide
Pr: Protein
P(AM-DMC): Poly(acrylamide [2-(methacryloyloxy)ethyl]-trimethylammonium chloride)
SRF: Specific resistance to filtration
SS: Suspended solids
TOC: Total organic carbon
TSS: Total suspended solids
YE: Yeast extract
